# Extended adjuvant endocrine treatment for premenopausal women: A Delphi approach to guide clinical practice

**DOI:** 10.3389/fonc.2022.1032166

**Published:** 2022-10-26

**Authors:** Giuseppe Buono, Grazia Arpino, Lucia Del Mastro, Alessandra Fabi, Daniele Generali, Fabio Puglisi, Alberto Zambelli, Saverio Cinieri, Francesco Nuzzo, Vincenzo Di Lauro, Paolo Vigneri, Giampaolo Bianchini, Filippo Montemurro, Alessandra Gennari, Michelino De Laurentiis

**Affiliations:** ^1^ Department of Breast and Thoracic Oncology, National Cancer Institute, Istituto di Ricovero e Cura a Carattere Scientifico (IRCCS) Fondazione “G. Pascale”, Naples, Italy; ^2^ Department of Clinical Medicine and Surgery, Oncology Division, University of Naples “Federico II”, Naples, Italy; ^3^ Department of Internal Medicine and Medical Specialties (DiMI), School of Medicine, University of Genova, Genoa, Italy; ^4^ Department of Medical Oncology, Istituto di Ricovero e Cura a Carattere Scientifico (IRCCS) “San Martino” General Hospital, Genoa, Italy; ^5^ Precision Medicine in Breast Cancer, Scientific Directorate, Department of Women Child and Public Health, Fondazione Policlinico Universitario A. Gemelli, Rome, Italy; ^6^ Breast Cancer Unit, Azienda Socio-Sanitaria Territoriale di Cremona, Cremona, Italy; ^7^ Department of Medicine, Surgery and Health Sciences, University of Trieste, Trieste, Italy; ^8^ Department of Medicine, University of Udine, Udine, Italy; ^9^ Department of Medical Oncology, Centro di Riferimento Oncologico (CRO) Aviano, National Cancer Institute, Istituto di Ricovero e Cura a Carattere Scientifico (IRCCS), Aviano, Italy; ^10^ Medical Oncology, “Papa Giovanni XXIII” Hospital, Bergamo, Italy; ^11^ Medical Oncology Division and Breast Unit, “Senatore Antonio Perrino” Hospital, Brindisi, Italy; ^12^ Department of Clinical and Experimental Medicine, University of Catania, Catania, Italy; ^13^ Department of Medical Oncology, Istituto di Ricovero e Cura a Carattere Scientifico (IRCCS) “San Raffaele” Hospital, Milan, Italy; ^14^ Breast Unit, Candiolo Cancer Institute, Fondazione del Piemonte per l’Oncologia - Istituto di Ricovero e Cura a Carattere Scientifico (FPO-IRCCS), Candiolo, Italy; ^15^ Medical Oncology, Department of Translational Medicine, University of Eastern Piedmont, Novara, Italy

**Keywords:** breast cancer, extended endocrine treatment, premenopausal patients, Delphi study, clinical and genomic risk, ovarian function suppression, aromatase inhibitors, tamoxifen

## Abstract

The use of an aromatase inhibitor (AI) in combination with ovarian function suppression (OFS) has become the mainstay of adjuvant endocrine therapy in high-risk premenopausal patients with hormone receptor-positive breast cancer. Although five years of such therapy effectively reduces recurrence rates, a substantial risk of late recurrence remains in this setting. Multiple trials have shown that extending AI treatment beyond five years could offer further protection. However, as these studies comprised only postmenopausal patients, no direct evidence currently exists to inform about the potential benefits and/or side effects of extended AI + OFS therapies in premenopausal women. Given these grey areas, we conducted a Delphi survey to report on the opinion of experts in breast cancer treatment and summarize a consensus on the discussed topics. A total of 44 items were identified, all centred around two main themes: 1) defining reliable prognostic factors to pinpoint premenopausal patients eligible for endocrine therapy extension; 2) designing how such therapy should optimally be administered in terms of treatment combinations and duration based on patients’ menopausal status. Each item was separately discussed and anonymously voted by 12 experts representing oncological institutes spread across Italy. The consensus threshold was reached in 36 out of 44 items (82%). Herein, we discuss the levels of agreement/disagreement achieved by each item in relation to the current body of literature. In the absence of randomized trials to guide the tailoring of extended AI treatment in premenopausal women, conclusions from our study provide a framework to assist routine clinical practice.

## 1 Introduction

Among women, breast cancer (BC) is the most common tumor worldwide, with an estimate of 19.3 million new diagnoses per year according to GLOBOCAN 2020 (1). Approximately 70% of BC cases are hormone receptor-positive (HR+) (2), so endocrine therapy (ET) has become the core of adjuvant treatment in this setting (3, 4). Several trials have proved the adjuvant endocrine therapy (AET) efficacy in reducing the recurrence rates of HR+ breast cancers during and after the first 5 years of treatment (5). However, women with endocrine-sensitive BCs retain a substantial risk of late recurrence, whose probability relates to the original clinical characteristics of the tumor (6, 7). Multiple randomized trials have thus recently emerged to evaluate both the benefits of extended (beyond 5 years) endocrine treatment (EET) in these patients and how such therapy should optimally be administered based on the available ET combinations (8–18). Of note, these studies have been conducted almost exclusively in postmenopausal women, leaving several grey areas for extended AET clinical practice in premenopausal patients. Closing this gap is pressing given that approximately 30% of BCs occur in premenopausal (<50 years) women (19), with inferior long-term outcomes in patients under age 40 years diagnosed with an HR+ disease (20, 21).

This age-related disparity depends, at least in part, on historical differences that have characterized the tailoring of the first 5 years of AET based on menopausal status. For all women with HR+ early breast cancer (EBC), aromatase inhibitors (AIs) are now a usual component of early adjuvant therapy, either instead of tamoxifen or in sequence with it as part of a switch therapy (22). However, tamoxifen monotherapy has remained the preferential treatment for premenopausal patients until recently, when a combined analysis of the SOFT and TEXT trials prompted a clinical shift in this setting (23). The study showed that, in premenopausal women, combining ovarian function suppression (OFS) with an AI is more beneficial in terms of disease-free survival (DFS) than a treatment with tamoxifen, either alone or alongside OFS (24–26). Notably, the benefits appear limited to patients with intermediate-to-high-risk clinicopathological features. On the contrary, the various treatments showed no relevant difference in low-risk patients, for whom 5 year tamoxifen monotherapy remains standard of care (26). Interestingly, the absolute benefit expected with AI + OFS seems comparable to that provided by chemotherapy (CT) in the TAILORx study (27). Originally designed to identify women with HR+ breast cancer who could be safely spared adjuvant CT based on their genomic risk of late recurrence, the TAILORx trial revealed that age significantly impacted the benefit of CT. More specifically, CT appeared to be beneficial only among patients with ≤50 years of age and a 21-gene recurrence score of 16-25 (28). Long-term results from a second trial, MINDACT, backed up this age-dependent benefit of CT, reporting an increasing absolute advantage of CT over time that was entirely attributable to the premenopausal group (29, 30). Data such as these, combined with the previous observation that a concomitant onset of amenorrhea in patients treated with CT correlated with better survival rates (31), led to hypothesize that chemotherapy beneficial effects in premenopausal women could be in part related to a CT-induced OFS (32, 33). Overall, these studies prompted international current guidelines to designate AI + OFS as the first-line strategy for the first 5 years of AET in premenopausal women with intermediate/high risk of relapse (5, 27).

As AET in younger patients has been modified, the need to optimize EET in the same setting has become compelling. To date, the effects of extending AI treatment beyond 5 years have been tested only in postmenopausal women, for which several randomized trials reported lower risks of BC recurrence and contralateral BC (10–17), particularly in node-positive patients (22, 34). In premenopausal patients, only the benefits of a 10-year tamoxifen monotherapy have been directly tested (8, 9). However, a recent secondary analysis of the SOFT and TEXT trials suggested that an extended AI treatment could be beneficial in this setting (27, 35). This study investigated the ability of different ET to prevent distant recurrences in a cohort of premenopausal women with HR+ disease, with a median follow-up of nine years in TEXT and eight years in SOFT. In this analysis, seven classic clinicopathologic features [age, tumor size, nodal status, grade, estrogen receptor (ER) level, progesterone receptor (PgR) level and Ki-67 expression level] were combined into a single continuous value named composite risk (i.e., the Regan risk score), which functions as a tool to stratify patients according to their differences in outcomes between various ET combinations (35). For high-risk patients (35 to 39 years of age, grade 3, pT2pN1a, ER and PgR of 50% or greater, Ki-67 26% or greater; composite risk, 3.00), the results revealed a direct correlation between the risk of recurrence and the benefit offered by 5 years of AI + OFS. Intermediate-risk patients (40 to 44 years of age, grade 2, pT1pN1a, ER and PgR of 50% or greater, Ki-67 of 20% to 25%; composite risk, 1.78) still benefited from AI + OFS. Low-risk patients (40 to 44 years of age, grade 2, pT1pN0, ER and PgR of 50% or greater, Ki-67 of 14% to 19%; composite risk, 0.89), on the contrary, did not show significant differences among the various treatments.

Of note, the above-mentioned data raise the question whether EET patients need to be specifically selected (i.e., based on high risk for late distance recurrence). In this respect, toxicity is a primary concern when contemplating EET. Depending on the administered drug, an extension of endocrine treatment could be associated with increased risk of uterine cancer and thromboembolic and cardiovascular events, decreased bone health, hot flashes, sexual dysfunction, and depression (24, 36–38).

In the absence of compelling evidence that can guide EET tailoring in premenopausal women, our study implemented a Delphi model to gain consensus on how to identify patients who could benefit from EET and, if so, how to optimally administer such treatment.

## 2 Material and methods

The Delphi approach applied to this study consisted of two phases. The first phase began in January 2020, when a Steering Committee (SC) reviewed all available published data on EET and defined two relevant topics on the subject at hand (1): prognostic features to identify patients eligible for EET and (2) EET therapeutic strategies in HR+ BC patients depending on menopausal status at the end of the first 5 years of AET. The SC then formulated a series of statements related to these topics and compiled them into a Delphi survey. In the second phase, the survey was extended to a panel of five Italian oncologists, using a web-based form due to the onset of the COVID-19 pandemic.

Overall, the consensus survey was completed by 12 experts in breast cancer treatment (seven members of the SC and five additional oncologists, hereinafter referred to as the Panel), representing oncological institutions distributed across the Italian territory.

Upon statements finalization, a survey was submitted and voted by the Panel. According to the Delphi method, the panelists were invited to vote anonymously on each statement using a five-point Likert scale defined as: 1 = completely disagree; 2 = partially disagree; 3 = partially agree; 4 = agree; 5 = completely agree (39). For each statement, a consensus was achieved if either the sum of answers 1 and 2 (disagreement) or that of answers 3, 4, and 5 (agreement) exceeded 66%, as previously described (40, 41).

All data were analyzed using descriptive statistics.

## 3 Results

During the first phase of the Delphi survey, the SC addressed the two defined topics and drafted a total of 19 statements, 11 for the first topic (patient eligibility for EET based on their clinicopathological and genomic features) and eight for the second one (EET therapeutic choices based on current patients’ menopausal status). As some of the statements included sub-options, the number of items to be separately voted totaled 44. **Tables 1**, **2** illustrate all generated items for the two topics.

**Table 1:** Level of agreement/disagreement and consensus status on statements regarding prognostic features for identifying patients eligible to extended endocrine treatment (EET).

Statements	Level of agreement/disagreement (%)					Consensus*
	**Completely disagree**	**Partially disagree**	**Partially agree**	**Agree**	**Completely agree**	
1) To identify patients eligible for EET, the following factors should be considered:						
a) Age	0%	20%	50%	10%	20%	**20% - 80%**
b) Tumor dimension (T)	0%	18%	0%	55%	27%	**18% - 82%**
c) Nodal status (N)	0%	0%	0%	8%	92%	**0% - 100%**
d) ER and PgR expression levels	0%	45%	0%	45%	10%	**45% - 55%**
e) Tumor grading	0%	42%	16%	42%	0%	**42% - 58%**
f) HER2 overexpression	0%	36%	28%	36%	0%	**36% - 64%**
g) Ki-67	8%	25%	25%	17%	25%	**33% - 67%**
h) Histology	0%	8%	17%	67%	8%	**8% - 92%**
i) Genomic test scores (Oncotype Dx, Prosigna, MammaPrint dx, EndoPredict, BCI)	0%	0%	43%	57%	0%	**0% - 100%**
l) Clinical scores (Composite risk (Regan score) and clinical treatment score (CTS5))	0%	25%	8%	67%	0%	**25% - 75%**
2) The involvement of axillary lymph node is not sufficient to offer patients EET.	8%	50%	17%	17%	8%	**58%** - **42%**
3) EET should be considered for patients with axillary nodal metastasis:						
a) regardless of the number of lymph nodes involved (N+)	25%	33%	33%	9%	0%	**58%** - **42%**
b) only if four or more lymph nodes are involved (N≥4)	33%	42%	17%	8%	0%	**75% - 25%**
4) A high clinical risk is sufficient to recommend EET.	0%	9%	36%	55%	0%	**9% - 91%**
5) The following clinical scores may be used to decide whether to recommend EET:						
a) Composite risk (Regan score)	9%	18%	46%	27%	0%	**27%** - **73%**
b) Clinical treatment score (CTS5)	0%	18%	46%	27%	9%	**18%** - **82%**
6) A high genomic risk is sufficient to recommend EET.	11%	11%	45%	33%	0%	**22%** - **78%**
7) The following genomic signatures may be used to decide whether to recommend EET:						
a) Oncotype DX™	75%	17%	8%	0%	0%	**92%** - **8%**
b) Prosigna®	17%	8%	8%	59%	8%	**25%** - **75%**
c) MammaPrint dx®	55%	18%	27%	0%	0%	**73%** - **27%**
d) EndoPredict® (Epclin)	17%	8%	25%	50%	0%	**25%** - **75%**
e) Breast Cancer Index™ (BCI)	18%	8%	33%	33%	8%	**26%** - **74%**
8) Patients with low clinical risk based on Regan score do not benefit from EET.	0%	8%	67%	25%	0%	**8%** - **92%**
9) Patients with low clinical risk based on CTS5 do not benefit from EET.	0%	27%	55%	18%	0%	**27%** - **73%**
10) Patients with low clinical risk, but an otherwise high genomic risk, might benefit from EET.	8%	0%	42%	50%	0%	**8%** - **92%**
11) Patients with high clinical risk, but an otherwise low genomic risk, might benefit from EET.	0%	17%	58%	25%	0%	**17%** - **83%**

Percentage in blue indicates the **level of agreement**, and in red the **level of disagreement**. *Consensus on agreement was reached if total levels of agreement (partial + complete) were >66%, and it is highlighted with a light blue background. Consensus on disagreement wasreached if total levels of disagreement (partial + complete) were >66%, and it is highlighted with a light red background. Consensus was not met if total levels of both agreement anddisagreement were ≤66%.

Consensus on agreement was reached in 27 out of 44 (61%) items, whereas nine (21%) items achieved a consensus on disagreement. Thus, 36 options (82%) succeeded while eight (18%) failed to get the consensus threshold among all participants.

### 3.1 Consensus levels on prognostic features for identifying patients eligible to EET

**Table 1** shows the levels of agreement/disagreement and consensus outcomes on items regarding patient eligibility for extended AET.

Only five items out of 26 (19%) did not reach a consensus, while the remaining 21 achieved a consensus on either agreement or disagreement (18 and 3 items, respectively).

The main area of debate among the panelists concerned the prognostic relevance of three clinicopathological features in defining which patients could benefit from EET. More precisely, expression levels of both ER and PgR, tumor grading, and overexpression of the human epidermal growth factor receptor 2 (HER2) reached levels of agreement equal to 55%, 58%, and 64%, respectively (**Table 1**, items 1.d, 1.e, and 1.f).

Rates of agreement/disagreement did not reach the consensus threshold (42% and 58%, respectively) also with regards to statement 2, which addressed whether the involvement of axillary lymph nodes is sufficient to justify EET. In line with this result, the Panel did not agree also on item 3.a, which asked whether EET is required in the presence of axillary nodal metastasis, irrespectively of the number of nodes involved.

Although the Panel agreed that the expression of the proliferative marker Ki-67 should be considered for risk assessment, the consensus on agreement for this feature was just above the threshold (67%; **Table 1**, item 1.g). For all the other items included in the survey, levels of agreement or disagreement among the panelists were relatively high, ranging from 73 to 100%.

### 3.2 Consensus levels on EET therapeutic choices in pre/perimenopausal and postmenopausal patients

Our study implemented a Delphi model to gain consensus on the extended AET strategies that could be considered for women who were premenopausal at the time of entering the first 5 years of AET. In doing so, the panelists were asked to express their opinions based on both the type of AET the patients received during the first 5 years and their menopausal status at the end of that period. Both aspects are of great relevance when evaluating the type of EET to offer. Indeed, while AIs are effective in postmenopausal women when provided alone, in younger patients they must be used only in combination with OFS to prevent them from causing negative hypothalamic feedback and ovarian stimulation (42). The Panel acknowledged that patients’ menopausal status at the end of the first 5 years of AET must be assessed by evaluating age, presence or lack of menstrual cycle, and hormone levels after suspending OFS. It was also agreed that perimenopausal patients who are not undoubtedly classifiable as postmenopausal should be treated as if they were premenopausal.

**Table 2** summarizes the level of agreement/disagreement and consensus on EET therapeutic choices with respect to patients’ menopausal status.

**Table 2:** Level of agreement/disagreement and consensus status on statements concerning the type and duration of extended adjuvantendocrine treatment (EAET).

Statements	Level of agreement/disagreement (%)					Consensus*
	**Completely disagree**	**Partially disagree**	**Partially agree**	**Agree**	**Completely agree**	
12) For patients treated with tamoxifen + OFS for 5 years who remain **pre/perimenopausal** at the end of that period, the following EET options might be considered:						
a) Tamoxifen for 5 years	0%	17%	8%	67%	8%	**17%** - **83%**
b) Tamoxifen + OFS for 5 years	36%	36%	10%	18%	0%	**72%** - **28%**
c) AIs + OFS for 5 years	27%	9%	55%	9%	0%	**36%** - **64%**
13) For patients treated with exemestane + OFS for 5 years who remain **pre/perimenopausal** at the end of that period, the following options for EET might be considered:						
a) Exemestane + OFS for 2-3 years	9%	27%	37%	27%	0%	**36%** - **64%**
b) Exemestane + OFS for 5 years	50%	25%	25%	0%	0%	**75%** - **25%**
c) Tamoxifen for 5 years	0%	25%	25%	42%	8%	**25%** - **75%**
14) For patients treated with tamoxifen + OFS for 5 years who become **postmenopausal** at the end of that period, the following EET options might be considered:						
a) Tamoxifen for 5 years	16%	17%	58%	9%	0%	**33%** - **67%**
b) AIs for 5 years	0%	8%	33%	26%	33%	**8%** - **92%**
15) For patients treated with exemestane + OFS for 5 years who become **postmenopausal** at the end of that period, the following EET options might be considered:						
a) Exemestane for 2-3 years	0%	18%	27%	46%	9%	**18%** - **82%**
b) Exemestane for 5 years	0%	0%	67%	25%	8%	**0%** - **100%**
c) Tamoxifen for 5 years	17%	75%	0%	8%	0%	**92%** - **8%**
16) For patients treated with tamoxifen + OFS for 2-3 years and then with AIs + OFS for additional 3-2 years (switch strategy) who become **postmenopausal** at the end of that period, the following EET options might be considered:						
a) AIs for 2-3 years	0%	8%	17%	75%	0%	**8%** - **92%**
b) AIs for 5 years	8%	0%	67%	25%	0%	**8%** - **92%**
17) For patients treated with exemestane + OFS for 5 years who experience low tolerability to exemestane, the following EET options might be considered:						
a) A different AI for 5 years	58%	25%	17%	0%	0%	**83%** - **17%**
b) Tamoxifen for 5 years	18%	0%	9%	46%	27%	**18%** - **82%**
c) Intermittent AI for 5 years	25%	42%	17%	16%	0%	**67%** - **33%**
18) In association with OFS, the three AIs anastrozole, letrozole and exemestane have the same efficacy and they can be interchangeably used in **pre/perimenopausal** women during both the first 5 years of AET and EET.	67%	8%	17%	8%	0%	**75%** - **25%**
19) For patients treated with exemestane + OFS for 5 years and subject to prophylactic bilateral mastectomy, EET for other 2-5 years might be considered.	25%	17%	25%	25%	8%	**42%** - **58%**

Percentage in blue indicates the **level of agreement**, and in red the **level of disagreement**. *Consensus on agreement was reached if total levels of agreement (partial + complete) were >66%, and it is highlighted with a light blue background. Consensus on disagreement wasreached if total levels of disagreement (partial + complete) were >66%, and it is highlighted with a light red background. Consensus was not met if total levels of both agreement anddisagreement were ≤66%. Bold value is to visually underline the differences between statements concerning pre/perimenenopausal and post-menopausal women.

For women who remained pre/perimenopausal after the first 5 years of AET, the Panel noted that a 5-year tamoxifen monotherapy could be chosen irrespectively of the treatment dispensed before (**Table 2**, item 12.a and 13.c). Moreover, for patients treated with tamoxifen + OFS in the first 5 years, the option of providing additional 5 years of the same combination achieved 72% of negative consensus, while no consensus was deemed for a 5-year extended therapy with AI + OFS (**Table 2**, items 12.b and 12.c). In case of women who had received 5 years of exemestane + OFS, the Panel reached no consensus on prolonging such treatment for 2-3 years and a negative consensus (75%) about an extension of 5 years (**Table 2**, items 13.a and 13.b, respectively).

As for women determined to be postmenopausal at the end of the first 5 years of AET, the Panel agreed that a therapy with AIs for additional 2-3 or 5 years was always suitable, irrespectively of the treatment previously administered (**Table 2**, items 14.b, 15.a, 15.b, 16.a, and 16.b). In this setting, an extended therapy with tamoxifen alone reached a consensus on agreement - although very mild (67%) - for patients treated with tamoxifen + OFS in the first 5 years and a high consensus on disagreement (92%) for those who had previously received a 5-year treatment with exemestane + OFS (**Table 2**, items 14.a and 15.c, respectively).

The panel also acknowledged that in case of low tolerability to exemestane during the first 5 years of AET, the intermittent or continuous use of a different AI for additional 5 years should be avoided (83% and 67% consensus on disagreement, respectively) in favor of a tamoxifen monotherapy (82% consensus on agreement) (**Table 2**, statement 17). These results applied to both pre/perimenopausal and postmenopausal patients.

Concerning which AI could be used in combination with OFS for the treatment of pre/perimenopausal women during both the first 5 years of AET and EET, the Panel disagreed that anastrozole, letrozole, or exemestane have the same efficacy in this setting (**Table 2**, statement 18, 75% consensus on disagreement).

Finally, no consensus was reached on whether EET should be offered to patients that had performed prophylactic bilateral mastectomy (**Table 2**, statement 19).

## 4 Discussion

Due to the results of the SOFT and TEXT trials, the combination AI + OFS has become the preferential strategy in the first 5 years of adjuvant treatment of intermediate-to-high-risk premenopausal patients with HR+ EBC (5, 22, 24–26, 43), while a tamoxifen monotherapy remained the standard of care in low-risk patients. To date, however, no compelling evidence exists to indicate if and how this type of therapy could be safely extended. Answering this question is pressing, as HR+ tumors retain a substantial risk of late recurrence after the initial AET (6). Randomized trials investigating the effects of extended AI treatments have been conducted in postmenopausal populations (22) but these data cannot be directly implemented in younger patients. In premenopausal women, AIs must only be used in combination with OFS (42), for which no evidence of benefits beyond 5 years of treatment is currently available. The Delphi approach used in this study identified and discussed two crucial topics for EET tailoring in younger women: how to detect high-risk patients who would benefit from extended AET and how to administer such treatment. The study also quantifies the levels of agreement/disagreement among panelists, who expressed their opinions based on professional experience and current literature. As most of the items discussed in our survey reached the consensus threshold, they may function as valuable recommendations for routine clinical practice.

### 4.1 Identification of pre/perimenopausal patients eligible for EET

In evaluating the absolute risk of late recurrence, different clinicopathological and genomic features have proved to be relevant and thus used to determine which patients might benefit from extended AET.

#### 4.1.1 Prognostic value of tumor- and patient-related features

Three tumor-related characteristics did not reach the consensus threshold, namely the expression levels of ER and PgR, tumor grading, and HER2 overexpression (**Table 1**, items 1.d, 1.e, and 1.f, respectively). On the first factor, the Panel noted that the following reasoning has led to non-conclusive voting. On one hand, tumors with lower levels of ER expression pose a greater risk of late recurrence than those with high expression, as pointed out in the current guidelines for AET in postmenopausal women (22). On the other hand, breast cancers with thresholds of ER-positivity lower than 10% behave similar to triple-negative BC, thus limiting the potential benefit of endocrine therapy (44).

As for the prognostic value of tumor grading, contrasting published data exist. A recent meta-analysis of the results of 88 trials involving women with estrogen receptor-positive (ER+) BC identified higher tumor grades as moderately predictive of the risk of distant recurrence during years 5 to 20 (rate ratio of low vs. high grade = 0.50, 95% CI: 0.37-0.67, p <0.001) (6). Accordingly, current guidelines on extended AET in postmenopausal women consider the prognostic value of tumor grading sufficiently robust to inform clinical decisions, although none of the studies of EET has stratified patients by this parameter so far (22). Nonetheless, a recent randomized trial in postmenopausal women reported no difference in DFS according to tumor grade when extending letrozole therapy for 5 years versus the standard duration of 2-3 years (both administered after 2-3 years of tamoxifen) (histological grade G1: hazard ratio [HR] = 0.837, 95% CI: 0.479-1.466; G2: HR = 0.797, 95% CI: 0.627-1.014; G3: HR = 0.795, 95% CI: 0.537-1.176) (13). Moreover, a retrospective analysis involving Danish women diagnosed with EBC between 1987 and 2004 reported no association of late recurrence with tumor grade (45). However, when stratifying the analysis by year, the findings for patients diagnosed after 2002 appeared to align with those reported in the above-mentioned meta-analysis (6), although these estimates might have been partly imprecise, as stated by the authors (45).

The lack of consensus on the prognostic relevance of HER2 overexpression mainly depends on the absence of randomized trials specifically addressing the benefits of EET in women with HR+/HER2+ breast cancers. The meta-analysis aforementioned analyzed also the association of HER2 with patients’ outcomes and defined the status of HER2 as not predictive (6). In this study, women with HER2+ tumors who did not receive trastuzumab-based chemotherapy had a worse prognosis during the first 5 years (but not thereafter) than those with HER2-negative tumors. Importantly, the authors hypothesized that a wider use of trastuzumab in HER2+ disease might have improved prognosis after 5 years, since most of the reduction in the recurrence rate with chemotherapy seems to occur during that time (46). As only 2% of the patients involved in this meta-analysis received trastuzumab, the authors could not evaluate this hypothesis, which was recently addressed by a combined analysis of the NCCTG N9831 and NRG Oncology/NSABP B-31 trials (47). This study demonstrated no significant difference during years 5-10 in the outcome between HR+/HER2+ and HR-/HER2+ breast cancers treated with adjuvant trastuzumab (HR = 1.62, 95% CI: 0.97-2.71, p = 0.065), although a higher risk of recurrence was assessed in patients with HR+/HER2+ BC and N3 disease (adjusted HR = 4.39, 95% CI: 0.94-20.50, p = 0.06).

Our survey revealed a very mild consensus on agreement (67%) for the prognostic value of the proliferative marker Ki-67, with positive votes almost equally allocated between partial agreement (25%), agreement (17%), and complete agreement (25%) (**Table 1**, item 1.g). The Panel recognized that, overall, these figures mirror the current level of uncertainty on the issue. According to the same meta-analysis of 88 trials mentioned previously, patients with a Ki-67 antibody expression ≥20% presented with higher risks of distant recurrence and death during the first 5 years (HR = 1.56, CI: 1.40-1.74 and HR = 1.65, C: 1.43-1.91, respectively), while this factor became of modest relevance in the years 5-20 (HR = 1.24, CI: 1.05-1.46 and HR = 1.23, CI: 0.99-1.53, respectively) (6).

High levels of agreement were achieved by factors like patient age, tumor dimension, nodal status, tumor histology, genomic test scores, and clinical scores (**Table 1**, items 1.a, 1.b, 1.c, 1.h, 1.i, and 1.l, respectively). As for patient-related features, younger age has been considered a poor prognostic value in HR+ tumors due to different reasons, including suboptimal estrogen inhibition, inconstant adherence to endocrine therapy, and age-related biologic differences in the tumors (48–52). In our survey, 80% of the total panelists considered age as a relevant factor in risk assessment, although most of them (50%) agreed only partially with the proposed statement (**Table 1**, item 1.a). This prevalence of partial agreement may be explained by the presence of conflicting data in the scientific literature. A meta-analysis that analyzed age association with local recurrence in women with EBC demonstrated that young age is a significant risk factor (recurrence rate [RR] = 2.21, 95% CI: 1.62-3.02) within 5 years of breast-conserving therapy. Yet, the same study failed to prove a significant association at 10 years (RR = 1.47, 95% CI: 0.96-2.27) (53). A second study revealed an increased risk of late BC recurrences in women aged < 40 years (HR = 1.47, CI: 1.22-1.78), although the effect was lost when considering all premenopausal patients (HR = 1.04, CI: 0.89-1.23) (45). As direct evidence is currently missing to prove that young age represents a risk *per se*, the Panel argued that this factor should be evaluated together with other prognostic features. Consistently, the Clinical Treatment Score post-5 years (CTS5), which integrates the risk factors of tumor size, nodal status, grade, and age, has proved to be a prognostic factor for late disease relapse in both pre- and postmenopausal women (54).

Tumor histology deemed a high grade of positive consensus (92%, **Table 1**, item 1.h). As argued by the panelists, different histopathological features are associated with variations in prognosis as well as in treatment response (55), so much so that cancer histology should be considered when evaluating the potential benefits of EET. Invasive ductal carcinoma (IDC) and invasive lobular carcinoma (ILC) of the breast, for instance, are associated with a worse prognosis when compared with other histopathological subtypes, such as tubular and mucinous carcinoma (56). Moreover, a competing risk model has recently shown that ILC strongly associates with the risk of developing contralateral breast cancer (57, 58). Therefore, panelists agreed that EET could be considered for patients with IDC and ILC, while it could be safely forgo in the case of histopathological subtypes associated with good prognosis if the patient is defined as low-risk based on other clinico-pathological features.

Our survey reached very high levels of agreement (82% and 100%, respectively) on considering both tumor dimension and nodal status as relevant predictors of late recurrence (**Table 1**, items 1.b and 1.c), in line with several published evidence that validated the prognostic value of these two histological factors. According to the same meta-analysis mentioned previously, the risk of distant recurrence for ER+ breast cancer strongly correlated with the initial tumor dimension and nodal status, ranging from 13% for T1N0 patients to 42% for T2N4-9 patients (6). Similarly, a Danish study published this year showed a cumulative incidence of disease relapse at 10-25 years, ranging from 12.7% for T1N0 to 24.6% for T2N4-9 patients (45). Moreover, the Early Breast Cancer Trialist Collaborative Group meta-analysis presented at the 2018 San Antonio Breast Cancer Symposium showed that nodal involvement could predict AI extension benefit (34). According to this analysis, the absolute benefit of a 5-year AI treatment after 5 years of AET ranged from 1.1% (HR = 0.82, CI: 0.71-0.95) in N0 disease to 7.7% (HR = 0.71, CI: 0.56-0.89) in a cohort of N4+ patients.

It is relevant to state that expert panels involved in three independent studies recommended that women with node-positive and HR+ breast cancer receive EET. Importantly, while in one case the recommendation was based on the review of evidences from six trials involving only postmenopausal women (22), the other two studies evaluated both benefits and risks related to premenopausal patients (59, 60). Remarkably, although our study achieved a complete agreement on the relevance of nodal status, it did not reach a consensus on statement 2, which addressed whether axillary lymph nodal involvement is *sufficient* to candidate patients to EET. No consensus was also reached when evaluating whether EET should be considered in the presence of axillary nodal metastasis *regardless of the number* (**Table 1**, item 3.a), while consensus on disagreement (75%) was met on recommending EET *only* to patients with ≥4 nodes involved (**Table 1**, item 3.b). Overall, these figures reflect the Panel’s suggestion to evaluate nodal involvement in the context of other clinicopathological parameters. Accordingly, the panelists agreed that high clinical risks based on either Regan risk score or CTS5 are not only relevant but also sufficient to identify patients who could benefit from EET (**Table 1**, item 1.l and statements 4 and 5). It is of relevance that both clinical scores integrate different risk factors, including nodal status. More specifically, the Regan score combines seven classic clinicopathologic features (age, tumor size, nodal status, grade, estrogen receptor level, progesterone receptor level, and Ki-67 expression level) into a single continuous value that represents differences in outcomes between various ET combinations. As for CTS5, it stratifies women after 5 years of AET into one of three recurrence risk groups (low, intermediate, and high risk) at 5 to 10 years.

#### 4.1.2 Prognostic value of clinical and genomic risks

The Panel agreed that patients with a low clinical risk, as determined by either Regan score or CTS5, do not benefit from EET, with the former clinical score reaching a stronger consensus on agreement compared with the latter (92% vs. 73%, respectively) (**Table 1**, statements 8 and 9). These results were grounded on several considerations. A secondary analysis of the SOFT and TEXT trials used the Regan risk score to identify premenopausal patients more likely to benefit from enhanced ET (27, 35). Absolute treatment effects were investigated across the continuum of composite risk for women with HR+/HER2− breast cancer, with a median follow-up of nine years in TEXT and eight years in SOFT. In brief, the study revealed that, while high-risk patients benefited more from an AI + OFS therapy than from a treatment with tamoxifen + OFS or tamoxifen alone (10% to 15% absolute improvement in 8-year freedom from distant recurrence), in low-risk patients the benefits of escalating ET were minimal (27, 35). With regard to CTS5, it was validated in two clinical trials (ATAC and BIG I-98) involving postmenopausal women, showing to be highly prognostic (61). However, a recent validation of the CTS5 among women with node-negative cancers enrolled in the TAILORx study showed that its prognostic value was weaker in premenopausal women (62). Moreover, only a minority of those younger women received OFS (28), thus limiting direct evidence of CTS5 performance in premenopausal patients who receive OFS-based therapy.

Importantly, the Panel agreed that patients with low clinical risk might still benefit from EET if their genomic risk was high (**Table 1**, statement 10), thus implying that genomic testing, if available, should be included in the decision-making process. The genomic risk is an individualized estimate of both distant recurrence risk and likelihood of CT benefit based on the expression levels of a certain number of breast cancer-related genes. It can be derived using one of the genomic tests currently available, including Oncotype DX™, Prosigna^®^, MammaPrint dx^®^, EndoPredict^®^ and Breast Cancer Index™ (BCI) (63–68). The Panel acknowledged that genomic signatures have not been specifically developed to estimate late distant recurrences and, to date, none of the studies on EET has stratified patients by genomic markers. Yet, retrospective findings on the relevance of genomic signatures have been considered sufficiently robust to inform the clinical decision about extended AET in postmenopausal women (22, 69). As such, our survey reached a positive consensus on considering genomic tests relevant and sufficient to identify premenopausal patients with a greater likelihood of benefiting from EET (**Table 1**, item 1.i, and statement 6). However, as direct evidence in this respect is currently missing, two important aspects must be emphasized at this point.

Firstly, none of the panelists *completely* agreed with statements 4, 6, 10, and 11, which assessed whether to rely on clinical or genomic scores when they are used alone or in the event of patients showing low genomic risk, but an otherwise high clinical risk, and vice versa. Secondly, the Panel recommended the use of Prosigna^®^, EndoPredict^®^, and BCI™ (consensus on agreement of 75%, 75%, and 74%, respectively) over Oncotype DX™ and MammaPrint dx^®^ (consensus on disagreement of 92% and 73%, respectively) (**Table 1**, statement 7). The leading causes for these figures are the results of a retrospective analysis that compared the prognostic value of six signatures for late distant recurrence (years 5 to 10) to investigate the potential value of EET (69). The study analyzed the prognostic power of four genomic signatures (Oncotype DX™, Prosigna^®^, BCI™, and EndoPredict^®^) and two clinical scores (Clinical Treatment Score (CTS) and 4-gene immunohistochemical score (IHC4)) in two populations of postmenopausal patients with ER+ breast cancer and either 0 or 1 to 3 positive nodes. In node-negative patients, the signatures Prosigna^®^ (HR = 2.77, 95% CI: 1.93-3.96), BCI™ (HR = 2.30, 95% CI: 1.61-3.30), and EndoPredict^®^ (HR = 2.19, 95% CI: 1.62-2.97) offered significantly more information than CTS alone (HR = 1.99, 95% CI: 1.58-2.50). On the contrary, adding Oncotype DX™ (HR = 1.69, 95% CI: 1.40-2.03) or IHC4 (HR = 1.95, 95% CI: 1.55-2.45) to CTS did not provide significant prognostic value. In the population with 1 to 3 positive nodes, all six signatures provided substantially less information, with EndoPredict^®^, Prosigna^®^ and BCI™ performing better than the other tests (HR=1.87, 95%CI 1.27-2.76; HR=1.65, 95%CI 1.08-2.51; and HR=1.60, 95%CI 1.04-2.47, respectively). Based on this retrospective analysis, the Panel raised two important points on the prognostic value of clinical and genomic risks for late distance recurrence: various signatures might differ, and the combination of clinical and molecular data may allow a more informed decision (particularly for node-positive cancers where each test alone seemed to provide limited independent information). Important to note is that, although the above-mentioned analysis did not include MammaPrint dx^®^, the Panel reached a 73% consensus on disagreement on the prognostic value of this genomic test (**Table 1**, item 7c). Central to the interpretation of this result is the fact that the same study compared the performance of the six different signatures also at 5 years after diagnosis. In this setting, all genomic tests provided independent information beyond CTS for women with both N0 and N1-3 disease. Taken together, the results of the study thus implied that the way a test performs might depend on its specific application (i.e., Oncotype DX™). Therefore, although MammaPrint dx^®^ was successfully used in the MINDACT trial to identify premenopausal patients who might safely forgo CT in favor of ET during the first 5 years of treatment, the Panel acknowledged that extrapolations on the prognostic value of MammaPrint dx^®^ for late recurrence should be made with caution, if ever, in the absence of definitive data.

### 4.2 Therapeutic strategy

In the second part of the survey, the panelists were requested to define what types of EET could be offered to patients on the basis of two main factors: the type of therapy provided in the first 5 years of AET and the patient’s menopausal status at the end of the initial treatment. To this end, eight independent statements were separately discussed and voted on (**Table 2**, statements 12-19). Herein, statements related to each menopausal status will be independently reviewed to facilitate the comprehension of the Panel debate on the available evidence.

#### 4.2.1 Therapeutic strategies in pre/perimenopausal patients

The first therapeutic issue addressed by the panelists was to define which EET could be suitable for women who remain pre/perimenopausal after 5 years of treatment with tamoxifen + OFS. The ATLAS and aTTom trials demonstrated that allocation to 5 more years of tamoxifen monotherapy is associated with an improvement in DFS of 3.7% (HR = 0.84, p = 0.002) and 4% (HR = 0.85, p = 0.003), respectively (8, 9). Importantly, the studies enrolled both pre- and postmenopausal patients with HR+ cancers, thus providing direct evidence of the beneficial effect of extended tamoxifen treatment in younger patients. Based on this evidence, the Panel agreed (83%) that an extended tamoxifen monotherapy could be safely used in this setting (**Table 2**, items 12.a). In contrast, the panelists did not recommend prolonging the treatment with tamoxifen + OFS for additional 5 years (72% consensus on disagreement), and they did not reach a consensus on the use of AI alongside OFS (**Table 2**, item 12.b and 12.c, respectively). Central to the discussion of this issue was the consideration that, to date, no direct evidence exists on the benefits of extended OFS therapy *per se*. On the contrary, while it was recognized that the long-term side effects of therapies such as tamoxifen or an AI in combination with OFS have not been well studied, the reported experience of another expert group is that patient tolerability of ET has been uniformly poor (33). Increased menopausal symptoms, sexual dysfunction, diabetes, and osteoporosis have been commonly described, for example, in patients receiving OFS (70). Of relevance, problems with long-term compliance with ET are especially important in younger patients, who will likely have to endure many years of therapy and its associated side effects (71).

The above-discussed concerns about OFS potential toxicity also grounded the debate around the second clinical issue addressed by the panelists, namely which EET could be suitable for women who received exemestane alongside OFS in the first 5 years of AET and remain pre/perimenopausal afterwards (**Table 2**, statement 13). A secondary analysis of the SOFT trial performed after eight years of median follow-up showed that the use of exemestane + OFS was associated with a significant benefit on DFS (HR = 0.65, CI: 95%, 0.53-0.81) compared with tamoxifen alone in the whole trial population, which included premenopausal women (35). Yet, the lack of direct evidence for long-term risks associated with extended OFS, combined with the above-mentioned findings of the ATLAS and aTTom trials on the benefits of extended tamoxifen monotherapy, prompted the Panel to reach a consensus on agreement (75%) only for 5 years of tamoxifen alone (**Table 2**, item 13.c). The options of extending exemestane + OFS for either 2-3 years or 5 years achieved no consensus or a consensus on disagreement, respectively (**Table 2**, items 13.a and 13.b).

Herein, it is of great importance to point out that at the time our Delphi survey was conducted, the results of two other consensus meetings had not been published yet (59, 60). During the recent 2021 St. Gallen/Vienna Consensus Conference on Early Breast Cancer Treatment Standards, a panel of international experts was asked to suggest how to treat high-risk patients who remained premenopausal after 5 years of tamoxifen + OFS. Among panelists who agreed to offer EET to these patients, 41% would recommend AI + OFS and 45% tamoxifen only (60). Moreover, another recent consensus study indicated that for selected premenopausal patients with high-risk node-positive disease, continuing OFS + tamoxifen or an AI may be considered (59). Had those outcomes been available for discussion, it is plausible to assume that our study might have achieved different results, especially for those items that did not get a consensus, such as items 12.c and 13.a. For instance, 55% of our panelists partially agreed to offer AI + OFS for years 5 to patients who had previously received tamoxifen + OFS (**Table 2**, items 12.c). In conclusion, although tamoxifen monotherapy appears as the safest option for EET in pre/perimenopausal women considering the current lack of data on extended AI + OFS therapy in this setting, the Panel acknowledges that there will be patients with sufficiently high recurrence risk to warrant the use of such treatment.

#### 4.2.2 Therapeutic strategies in postmenopausal patients

For patients who are determined to be postmenopausal after 5 years of tamoxifen + OFS, the consensus on agreement to extend tamoxifen monotherapy for other 5 years was just above the threshold (67%), with most of the panelists (58%) expressing only a partial agreement (**Table 2**, item 14.a). On the contrary, the option of offering 5 years of an AI achieved a high consensus on agreement (92%, **Table 2**, item 14.b). Central to these outcomes were the findings from different studies proving the benefits of extending therapy by switching from tamoxifen to an AI. In more detail, the MA.17 and NSABP B-33 trials showed that postmenopausal women can be safely switched to an AI after 5 years of tamoxifen. Improvements in DFS were equal to 4.6% (HR = 0.58, p < 0.001) and 2% (HR = 0.68, p = 0.07), respectively, among women who received extended 5 years of AI compared with those randomly assigned to placebo (10, 11). Moreover, a recent systematic review of such randomized trials noted that the relative benefits of EET appear most pronounced in women who switch from tamoxifen to an AI compared with those who continued with tamoxifen (22).

As for how to treat women who become postmenopausal after 5 years of exemestane + OFS, the option of offering tamoxifen monotherapy reached a strong consensus on disagreement (92%, **Table 2**, item 15.c), while exemestane monotherapy obtained high levels of positive consensus, irrespectively of the duration of the treatment (82% for 2-3 years and 100% for 5 years; **Table 2**, items 15.a and 15.b). Of note, the Panel strongly agreed on considering extended AI treatment beneficial for a postmenopausal population also after a switch strategy of 2-3 years of tamoxifen + OFS followed by 3-2 years of AI + OFS (**Table 2**, statement 16). Several considerations have allowed composing a rather uniform group recommendation on this issue, including the results of trials conducted on postmenopausal women who were offered additional ongoing AI therapy after receiving an AI upfront. More specifically, the MA 17-R trial compared an AI with a placebo for 5 years in women who had already received 4.5 to 6 years of adjuvant therapy with an AI, preceded in most cases by treatment with tamoxifen. Additionally, the NSABP B-42 study compared an AI with a placebo in women who had completed 5 years of endocrine therapy that consisted of either 5 years of an AI or up to 3 years of tamoxifen followed by an AI for a total of 5 years. In each of these trials there was a significant improvement in DFS for average-risk patients (4%, HR = 0.79, p = 0.01 for MA 17-R and 3%, HR = 0.79, p = 0.048 for NSABP B-42).

Concerning the question of how long the use of an AI could be prolonged for the treatment of women who become postmenopausal, the Panel strongly agreed that extensions of either 2-3 years or 5 years could both be offered (**Table 2**, items 15.a, 15.b, 16.a, and 16.b). Uniform recommendations on this issue were grounded on various considerations. The ABCSG-16 and IDEAL trials have compared shorter extended treatment durations versus longer durations (16, 72). In these studies, women received either 2 to 2.5 years or 5 years of extended treatment with an AI after an initial 5 years of AET. No difference was seen in disease recurrence rates, although the IDEAL study showed a numerically lower risk of recurrence after the separation of treatment arms at 2.5 years. Overall, these results offer some reassurance that treatment on the order of 7 to 8 years instead of 10 years does not appear to significantly compromise long-term outcomes in average-risk patients. Interestingly, the DATA trial conducted in postmenopausal women with HR+ breast cancer who were treated for 2-3 years with adjuvant tamoxifen showed that extended therapies with an AI for either 3 or 6 years were effective. The 5-year adapted DFS was 83.1% (95% CI: 80.0-86.3) in the 6-year group and 79.4% (76.1-82.8) in the 3-year group (HR = 0.79, 95% CI: 0.62-1.02, p = 0.066). Of note, patients in the 6-year treatment group had more adverse events than those in the 3-year treatment group, including arthralgia, myalgia, osteopenia, and osteoporosis (12). In line with the reasoning that shorter treatments might associate with reduced side effects compared with longer ones, the overall percentages of panelists that agreed or completely agreed with the option of a 2-3-year extended treatment with AIs were higher compared to those in favor of a longer therapy (**Table 2**, compare items 15.a to 15.b and 16.a to 16.b). Yet, the Panel still acknowledged that an overall treatment of 10 years could be considered for very high-risk patients (i.e., with T3-4, N3 disease).

#### 4.2.3 Other relevant aspects: AI selection in pre/perimenopausal women and EET in surgically treated patients

For patients of any menopausal status who presented low tolerability to exemestane during the first 5 years of AET, the Panel agreed (82% positive consensus) to offer additional 5 years of tamoxifen monotherapy, while it disagreed on the use of a different AI or of an intermittent dosing of exemestane (83% and 67% negative consensus, respectively) (**Table 2**, statement 17). This issue is particularly relevant in the case of pre/perimenopausal women, for which the panellists disagreed that the three AIs anastrozole, letrozole, and exemestane can be used interchangeably in association with OFS during both primary and extended AET (75% consensus on disagreement, **Table 2**, statement 18). These outcomes were mainly influenced by secondary analyses of the SOFT and TEXT trials that demonstrated the efficacy of an exemestane-based anti-estrogen therapy in premenopausal women. A joint analysis of these trials quantified the absolute treatment effect of the combination exemestane + OFS compared with tamoxifen + OFS or tamoxifen alone in premenopausal women, revealing an improvement ranging from 10 to 15% of the 5-year breast cancer-free interval (BCFI) for high-risk patients (73). The updated analysis of the SOFT and TEXT trials at nine years of median follow-up confirmed these data; the use of exemestane + OFS was associated with a 4% absolute improvement in 8-year DFS rate (HR = 0.77, 95% CI: 0.67-0.90, p < 0.001) (26). As for anastrozole, the ABCSG-12 trial compared the efficacy of this AI with that of tamoxifen in a population of premenopausal patients with ER+ EBC. Notably, the study revealed that, while there was no difference in DFS between patients treated with tamoxifen alone or anastrozole alone (HR = 1.08, 95% CI 0.81-1.44; p = 0.591), overall survival was worse using anastrozole than with tamoxifen (46 vs 27 deaths; HR = 1.75, 95% CI 1.08-2.83; p = 0.02). Given the above-mentioned data, the Panel acknowledged that exemestane could represent the preferential option for routine clinical practice. Yet, patients intolerant to exemestane could be offered an alternative AI. Indeed, although the efficacies of anastrozole, letrozole and exemestane were never directly compared in premenopausal women, the clinical responses to the three agents are similar in postmenopausal patients (74, 75). Importantly, special reference needs to be made for patients with elevated body mass index (BMI) when evaluating an alternative to exemestane.

A retrospective analysis of the ABSCG-12 trial revealed that overweight patients treated with anastrozole had a 60% increase in the risk of disease recurrence (HR = 1.60; 95% CI: 1.06-2.41; p = 0.02) compared with normal weight patients treated with the same AI. Moreover, in the overweight group, patients treated with anastrozole had a nearly 50% increase in the risk of disease recurrence (HR = 1.49; 95% CI: 0.93-2.38; p = 0.08) compared with those treated with tamoxifen (76). The results of a retrospective analysis of another trial, ABCSG-6a, strengthened the observation that an extended anastrozole treatment is not beneficial in postmenopausal women with high BMI (77). The study showed that, while additional 3 years of anastrozole halved the risk of disease recurrence compared with placebo in average weight patients (HR = 0.48; p = 0.02), overweight patients derived no benefit from extending anastrozole treatment for 3 years (DFS HR = 0.93, p = 0.68). Taken together, the above-discussed results suggest that BMI could significantly reduce the efficacy of anastrozole, probably due to a poorer suppression of aromatase activity and/or plasma estrogen levels. The latter were indeed proved to be higher in postmenopausal women with elevated BMI (78, 79). This limitation might be overcome using letrozole. The ALIQUOT study demonstrated that letrozole leads to a more complete suppression of plasma estradiol and estrone sulfate levels than anastrozole in a population of postmenopausal women with ER+ breast cancer (mean residual estradiol: 10% for anastrozole and 5.9% for letrozole; residual estrone sulfate levels; 4.6% for anastrozole and 2.0% for letrozole; p = 0.001) (80). A secondary analysis of this trial analyzed the correlations between estradiol and estrone sulfate levels in response to each AI and BMI, showing evidence of incomplete suppression by anastrozole in heavier women and of greater suppression with letrozole (81). Indeed, weaker, positive correlations were found between BMI and on-treatment estradiol and estrone sulfate levels with letrozole (*r* = 0.35, p = 0.013 and *r* = 0.30, p = 0.035, respectively) while, although showed a similar trend, they were not significant for anastrozole. While the latter finding would benefit from the study being repeated in a larger population, the Panel recognized that valid indications exist to recommend the use of letrozole instead of anastrozole in patients with intolerance to exemestane and elevated BMI.

The last statement discussed by the panelists addressed whether 2-3 years of EET should be offered to patients that performed prophylactic bilateral mastectomy. On this topic, opinions were split, with 42% disagreeing and 58% agreeing (**Table 2**, statement 19). The panelists who considered EET dispensable in this setting argued that, since a substantial fraction of improvement in DFS relates to secondary prevention of contralateral breast cancer, the prognosis in women who have undergone bilateral mastectomy may be sufficiently favorable to forego extended AI therapy, as also noted in the current guidelines for extended AET in HR+ breast cancers (22). In the opinion of the rest of the Panel, however, extended therapy in the setting of higher-risk breast cancers could provide a significant enough reduction in risk of distant recurrence to warrant the continuation of endocrine therapy.

## 5 Conclusion

At present, sufficient evidence is missing to allow a definitive recommendation for EET tailoring in women with HR+ EBC who were premenopausal when entering their initial therapy with AI + OFS. As such, our study used a Delphi approach to collect the opinions of experts in BC treatment on this uncertain topic and integrate such views into a framework that could assist routine clinical practice. In so doing, the panelists combined their extensive professional experience with extrapolations from first-hand results and secondary analyses of randomized clinical trials. As most of the addressed issues reached a consensus, statements in **Tables 1** and **2**, and their relative discussions, could guide EET therapeutic decisions in HR+ EBC pre/perimenopausal patients. Special reference needs to be made for those items in **Table 2** that address the extended use of AI + OFS in pre/perimenopausal women. It is realistic to believe that at least two of them would have reached a positive consensus – instead of no consensus - had the results of subsequently published studies been available at the time of our Delphi survey. Hence, despite the absence of first-hand data proving the benefits of extended AI + OFS treatment, the Panel recognises that such treatment could be offered to pre/perimenopausal patients with sufficiently high recurrence risk. It should also be acknowledged that novel empiric data cannot be produced when dealing with consensus studies and emphasized the urgent need of clinical trials assessing how to optimally provide extended AET in the setting considered in this study. Lastly, being the current analysis limited to the Italian setting, the Panel recommends caution when generalizing results, as resources and regulations might differ in other countries. As such, integrating the recommendations of our Panel with those published by other expert panels is advisable.

## Data availability statement

The raw data supporting the conclusions of this article will be made available by the authors, without undue reservation.

## Author contributions

Study conception and design, GBu and MDL. Identification of the topics and formulation of the statements, GBu, MDL, GA, AF, LDM, DG, FP, and AZ. Collection, interpretation, and discussion of data from the Delphi survey and literature, all authors. Manuscript drafting, GBu and MDL. Manuscript editing, all authors. All authors contributed to the article and approved the submitted version.

## Funding

The work was funded with a non-conditioning grant by Genetic S.p.a. The funder was not involved in the study design, collection, analysis, interpretation of data, the writing of this article or the decision to submit it for publication.

## Acknowledgments

We would like to thank Cecilia Grimaldi for writing and editorial assistance.

## Conflict of interest

GBu received honoraria or speakers’ fee from Novartis, GSK, Eli-Lilly, Pfizer, AstraZeneca, Roche, Daiichi Sankyo, Exact Science, Genetic. GA received consulting fees from Roche, Pfizer, Lilly, MSD, AstraZeneca and Novartis. LDM has received personal fees from Novartis, Pfizer, Roche, Eli Lilly, AstraZeneca, Pierre Fabre, Eisai, Daiichi Sankyo, Seagen, Gilead, Exact Sciences and Ipsen. AF received honoraria or speakers’ fee from Roche, Pfizer, Eli-Lilly, Novartis, Eisai, AstraZeneca, Exact Science, Epihonpharma, Daiichi-Sanlyo, Gilead, Seagen. DG received honoraria or speakers’ fee from Eli Lilly, Novartis, Pfizer, AstraZeneca, Roche and Eisai. FP received grants/research support from AstraZeneca, Eisai and Roche and the receipt of honoraria or consultation fees from Amgen, AstraZeneca, Daiichi Sankyo, Celgene, Eisai, Eli Lilly, Gilead, GSK, Ipsen, MSD, Novartis, Pierre-Fabre, Roche, Seagen, Takeda and Viatris, all disclosures are outside the submitted work. AZ reports personal fees and no-financial support from Novartis, AstraZeneca, Lilly, Pfizer, Daiichi Sankyo, MSD, Roche, Seagen, Exact Science, Gilead and Istituto Gentili, all disclosures are outside the submitted work. SC received honoraria or speakers’ fee from Eli Lilly. FN received speaking honoraria and travel grants from Istituto Gentili and Pfizer. VL received speaking honoraria and travel grants from Roche, Novartis, Lilly, Pfizer, Gilead, Seagen, Gentili, Takeda, Exact Science. PV received honoraria from AstraZeneca, Eli Lilly, Gilead, GSK, Istituto Gentili, Novartis, Pfizer and Teva. Research funding from Novartis and Pfizer. GBi received Consultancy/Honorarium fees from Roche, Pfizer, AstraZeneca, Lilly, Novartis, Neopharm Israel, Amgen, MSD, Chugai, Sanofi, Daiichi Sankyo, EISAI, Gilead, Seagen and Exact Science. FM received Consultancy/Honorarium fees from AstraZeneca, Eli Lilly, Roche, Novartis, Seagen, Pfizer, MSD, Daiichi Sankyo. AG received honoraria from AstraZeneca, Eli Lilly, Gilead, Istituto Gentili, Novartis, Pfizer, Seagen, Daiichi Sankyo, EISAI and Teva. MDL received honoraria or speakers’ fee from AstraZeneca, Amgen, Celgene, Daiichi Sankyo, Eisai, Eli Lilly, Exact Science, Gilead, MSD, Novartis, Pfizer, Pierre Fabre, Roche and Seagen.

## Publisher’s note

All claims expressed in this article are solely those of the authors and do not necessarily represent those of their affiliated organizations, or those of the publisher, the editors and the reviewers. Any product that may be evaluated in this article, or claim that may be made by its manufacturer, is not guaranteed or endorsed by the publisher.
